# Lesion size is associated with genetic polymorphisms in *TLR1*, *TLR6*, and *TIRAP* genes in patients with major abscesses and diabetic foot infections

**DOI:** 10.1007/s10096-019-03732-7

**Published:** 2019-11-30

**Authors:** Valentino D’Onofrio, Annelie A. Monnier, Cécile Kremer, Mark H. T. Stappers, Mihai G. Netea, Inge C. Gyssens

**Affiliations:** 1 Faculty of Medicine and Life Sciences, Research Group of Immunology and Biochemistry, Hasselt University, Hasselt, Belgium; 2 Department of Infectious Diseases and Immunity, Jessa Hospital, Hasselt, Belgium; 3 Department of Internal Medicine and Center for Infectious Diseases, Radboud University Medical Center, Nijmegen, The Netherlands; 4 Interuniversity Institute for Biostatistics and Statistical Bioinformatics, Hasselt University, Hasselt, Belgium; 5 Department of Biosciences, Geoffrey Pope Building, University of Exeter, Stocker Road, Exeter, EX4 4QD UK; 6 Human Genomics Laboratory, Craiova University of Medicine and Pharmacy, Craiova, Romania

**Keywords:** Abscess, Diabetic foot infection, Innate immunity, Single-nucleotide polymorphism, Pattern recognition receptors

## Abstract

Genetic variation in Toll-like receptors (TLRs) has previously been associated with susceptibility to complicated skin and skin structure infections (cSSSIs). The aim of this study was to investigate associations between the severity of cSSSIs, i.e., major abscesses and diabetic foot infections (DFIs), and a set of genetic polymorphisms in the Toll-like receptor pathway. A total of 121 patients with major abscesses and 132 with DFIs participating in a randomized clinical trial were genotyped for 13 nonsynonymous single-nucleotide polymorphisms (SNPs) in genes coding for TLRs and the signaling adaptor molecule TIRAP. Infection severity was defined by lesion size at clinical presentation for both types of infections. The PEDIS infection score was also used to define severity of DFIs. Linear regression models were used to study factors independently associated with severity. In patients with large abscesses, hetero- or homozygosity for the allelic variant *TLR6* (P249S) was associated with significantly smaller lesions while homozygosity for the allelic variant *TLR1* (R80T) was associated with significantly larger lesions. PRRs genes were not significantly associated with PEDIS. However, patients with DFI hetero- or homozygous for the allelic variant *TLR1* (S248N) had significantly larger lesions. Polymorphisms in *TLR1* and *TLR6* influence the severity of cSSSIs as assessed by the lesion size of major abscesses and DFIs. ClinicalTrial.gov Identifier: NCT 00402727

**Electronic supplementary material:**

The online version of this article (10.1007/s10096-019-03732-7) contains supplementary material, which is available to authorized users.

## Introduction

Skin and skin structure infections (SSSIs) refer to a large spectrum of clinical infectious syndromes involving the layers of the skin and its associated underlying soft tissues [1]. SSSIs can vary widely in presentation, etiology, and severity: ranging from mild infections, such as uncomplicated major abscesses, to life-threatening necrotizing fasciitis [1]. Characterizing features include redness, warmth, edema, and tenderness of the skin, typically paired with (purulent) discharge. The majority of SSSIs are caused by Gram-positive bacteria, predominantly *Staphylococcus aureus* and β-hemolytic streptococci. The management of complicated SSSIs (cSSSIs) typically involves both surgical debridement and empirical antibiotic therapy.

SSSIs are among the most commonly observed infections in clinical practice. However, determining the exact prevalence is challenging due to the diversity of clinical presentations. Furthermore, the incidence of SSSIs has been on the rise in the past years. The rate of clinically diagnosed SSSIs has risen to 500 episodes per 10,000 patient-years over the last decade [2].

Because of their frequent occurrence and low mortality, SSSIs are preferred clinical conditions included in registration studies for new antibiotics. In its 1998 guidance, the FDA*-*described cSSSIs as infections of the deeper soft tissue, involving surgical intervention or a significant underlying disease state complicating treatment (e.g., major abscesses, infected ischemic ulcers or diabetic foot infections, wounds, and burn infections) [3]. However, as per 2013, the FDA issued a new guidance with the term acute bacterial skin and skin structure infection (ABSSSI). These include large abscesses, wound infections, cellulitis, and erysipelas of at least 75 cm^2^ surface area [4]. In addition, in this new guidance, clinical response is defined as a ≥ 20% reduction in lesion size after 48 to 72 h compared to baseline [4].

When the skin is breached, cells of the innate immune system are able to recognize the invading bacteria through conserved essential structures of the microbes, called pathogen-associated molecular patterns (PAMPs), using specific pattern recognition receptors (PRRs). TLRs are a class of PRRs expressed by various immune cells including monocytes, macrophages, dendritic cells, neutrophils, NK cells, adaptive immune cells, and non-immune cells such as epithelial, endothelial, and stromal cells [5]. TLRs recognize PAMPs by N-terminal leucine-rich repeats, followed by a transmembrane region leading to the cytoplasmic Toll/interleukin-1R homology (TIR) domain, which interacts with several adaptor proteins, such as myeloid differentiation primary response gene 88 (MyD88) and MyD88 adaptor-like/Toll-interleukin 1 receptor domain-containing adaptor protein (TIRAP), activating transcription factors [6]. Recognition of PAMPs by TLRs results in the production of pro- and anti-inflammatory cytokines and the induction of an adaptive immune response [7]. Genetic variation, more specifically single-nucleotide polymorphisms (SNPs) in TLRs have previously been associated with susceptibility to cSSSIs [8, 9]. Polymorphisms in PRRs genes coding for TLR1, TLR2, and TLR6 are associated with an increased susceptibility to cSSSIs [8]. Interestingly, these particular TLRs are known to play a crucial role in the recognition of Gram-positive bacteria by the innate immune system [5]. In contrast, three polymorphisms in the *TLR10* gene were found to be associated with a decreased susceptibility to cSSSIs [9].

The aim of this study was to investigate associations between a set of *TLR* and signaling adaptor *TIRAP* gene polymorphisms and the severity of infection of patients with cSSSIs presenting to the hospital.

## Methods

### Study design

This study is a secondary analysis of the previously published RELIEF trial, which investigated the efficacy and safety of moxifloxacin for the treatment of SSSIs (clinicaltrial.gov identifier NCT 00402727) [10, 11]. The RELIEF study was a prospective, randomized, double dummy, double-blind, multinational, multicenter trial, involving adult men and women (≥ 18 years old) with a diagnosis of cSSSI with a duration of < 21 days that required hospitalization and initial parenteral antibiotic treatment for ≥ 48 h. Patients were enrolled between September 2006 and June 2008 in 61 centers, predominantly in Eastern Europe. Documented approval for the study was obtained from the Ethics Committee for all participating centers. Written informed consent was obtained from all participants.

### Study subjects

From a total population of 813 patients with cSSSI included in the RELIEF study, 389 patients gave informed consent to participate in the genetic substudy (supplementary Figure 1). Reasons for non-inclusion were lack of an invitation to participate in the genetic substudy or refusal to participate. Participants in the RELIEF study were geographically matched with Eastern European healthy controls. Therefore, of the 389 patients, 71 were excluded because they were not of European ancestry (*n* = 2) or not of Eastern European ethnicity (*n* = 69), resulting in 318 patients with cSSSI from 7 East European countries which were genotyped [8]. Patients with specific diagnoses were selected for this subanalysis, namely 121 patients diagnosed with major abscesses and 132 patients with diabetic foot infections (DFI).

### Definitions

Enrolment of patients was performed in compliance with the regulations available at the time of the trial [3]. CSSSIs included major abscesses and DFIs*.* Diagnoses were validated by an independent (and blinded for therapy) Data Review Committee (DRC) based on digital photographs of the infections. The following definitions were used [10]:

- Major abscesses were defined as collections of pus associated with extensive cellulitis, requiring antibiotic therapy in addition to surgical incision and drainage.
- DFIs were defined as infections occurring below the ankle in patients with confirmed diabetes. Severity of infection of DFIs was scored using the PEDIS (perfusion extent/size, depth/tissue loss, infection and sensation) infection grading system [12]. Only patients with mild to severe infection intensity (PEDIS grades 2–4) were included in the trial.
- Lesion size at baseline was considered a surrogate measure of severity of infection at presentation for both abscesses and DFIs in this analysis. Local extension of infection (abscess or DFI surrounded by cellulitis, i.e., erythema, induration, and edema) was indicated by the surface area (cm^2^) of the primary lesion. The surface area was calculated by multiplying the largest diameter by the second largest diameter measured perpendicularly to the first diameter with a flexible ruler. The area was measured by the investigators at baseline and validated by photographs provided to the DRC. Lesion size measurements have shown to be reliable and highly reproducible, regardless of definition in several studies [13].

### Genotyping

The method of genotyping is described by Stappers et al. In total, 13 nonsynonymous missense SNPs in genes coding for TLR1, TLR2, TLR4, TLR6, TLR 10, NOD2, and TIRAP were genotyped (supplementary Table 1) [8, 9].

### Selection of demographic and lifestyle factors

The selection of demographic- and lifestyle-related risk factors for major abscesses and DFI included in the analyses was based on literature. Demographic and lifestyle factors with the potential to influence immune function were chosen. Variables that could be confounders in the association between PRR SNPs and severity of infection included the demographic factors age, gender, BMI, the presence of comorbidities, and lifestyle factors smoking, and alcohol consumption. It is known that immune activity declines with age, resulting in less inflammation following infection [14]. Furthermore, many sex-related differences play a role in quantity of immune cells, cytokine production, and activity of granulocytes, which translates to a higher immune activity in women [15]. Previous studies have also shown an association between BMI and immune response with counts of leukocytes, neutrophils, and monocytes reported to be more elevated in persons with obesity [16]. In addition, smoking is associated with increased susceptibility to infections. Tobacco use affects neutrophil and monocyte functions [17]. Alcohol exposure decreases the immune systems’ first response by suppressing cytokine release and impairing phagocyte function [18]. Lastly, Hb1Ac and peripheral arterial disease were considered confounders in patients with DFIs.

### Statistical analysis

Descriptive statistics were used to analyse patients’ characteristics. Continuous data are reported as mean ± SD. Categorical variables are shown as number and proportion. As an explorative analysis, the univariate association between lesion surface area in patients with major abscesses or DFI as a continuous variable was investigated using the Kruskall–Wallis test (categorical) or Kendall’s tau (continuous).

Linear regression models were used to study factors associated with lesion surface area as a continuous variable. In both regression analyses, model selection was done in a stepwise backward manner based on significance level *p* < .05, starting from a full model including all factors. Risk was expressed as adjusted odds ratio (AOR) for logistic regression, with 95% CI. SNPs were evaluated using a dominant model analysis (in which heterozygous and homozygous individuals for the allelic variant are combined and compared to wild-type individuals) and a recessive model analysis (in which wild-type and heterozygous individuals are combined and compared to homozygous individuals for the allelic variant). Pairwise linkage disequilibrium (LD) for the different polymorphisms was calculated as described in Stappers et al. [8]. No correction for multiple testing was performed for the univariate analyses (shown in supplementary materials). SAS version 9.4 was used for all analyses.

## Results

Demographics and baseline characteristics of the abscess and DFI populations are shown in Table 1. Microbiological characteristics are shown in supplementary Table 2. The distribution of the genetic variants in PRRs and signaling adaptor *TIRAP* in patients is provided in supplementary Table 3. LD for the different polymorphisms was calculated as described in Stappers et al. LD analyses indicated that polymorphisms TLR1 S248N and TLR1 R80T (*p* = .001; *r*^2^ = 0.21; *D*′ = 0.40), TLR1 S248N and TLR6 P249S (*p* < .0001; *r*^2^ = 0.48; *D*′ = 0.59), TLR1 R80T and TLR6 P249S ( *p* < .0001; *r*^2^ = 0.27; *D*′ = 0.46), TLR1 R80T and TLR4 T399I (*p* < .007; *r*^2^ = 0.17; *D*′ = 0.26), and TLR1 R80T and TLR4 D299G (*p *< .007; *r*^2^ = 0.17; *D*′ = 0.26) were not independent from another, although the strength of their correlation was observed to be low. In contrast, LD analyses for TLR4 D299G and TLR4 T399I revealed that these SNPs were perfectly correlated (*p* < .0001; *r*^2^ = 1.00; *D*′ = 1.00). In addition, polymorphisms TLR10 I775L and TLR10 I369L (*p* < .0001; *r*^2^ = 0.58; *D*′ = 0.77), TLR10 N241H (*p* < .0001; *r*^2^ = 0.58; *D*′ = 0.77), TLR1 S248N (*p* < .0001; *r*^2^ = 0.69; *D*′ = 0.83), TLR2 R753Q (*p* = .04; *r*^2^ = 0.13; *D*′ = 0.80), and TLR6 P249S (*p* < .0001; *r*^2^ = 0.33; *D*′ = 0.47), TLR10 I369L and TLR10 M326T (*p* < .0001; *r*^2^ = 0.26; *D*′ = 0.61), TLR10 N241H (*p* < .0001; *r*^2^ = 1.00; *D*′ = 1.00), TLR1 S248N (*p* < .0001; *r*^2^ = 0.67; *D*′ = 0.80), TLR1 R80T (*p* < .0001; *r*^2^ = 0.32; *D*′ = 0.44), and TLR6 P249S (*p* < .0001; *r*^2^ = 0.58; *D*′ = 0.75), TLR10 M326T and TLR10 N241H (*p* < .0001; *r*^2^ = 0.27; *D*′ = 0.61), TLR1 R80T (*p* = .02; *r*^2^ = 0.15; *D*′ = 1.00), and TLR6 P249S (*p* = .01; *r*^2^ = 0.15; *D*′ = 0.60), and TLR10 N241H and TLR1 S248N (*p* < .0001; *r*^2^ = 0.67; *D*′ = 0.80), TLR1 R80T (*p* < .0001; *r*^2^ = 0.32; *D*′ = 0.44), and TLR6 P249S (*p* < .0001; *r*^2^ = 0.58; *D*′ = 0.75) were not independent from another, and strongly correlated to each other as well.

**Table 1:** Patient demographics and baseline characteristics of patient population with major abscesses (*n* = 121) or diabetic foot infections (DFI) (*n* = 132)

	Major abscesses (*n* = 121)	DFI (*n* = 132)
Age, mean (SD), years	47.6 (16.4)	60.4 (10.5)
Gender, male, *n* (%)	88 (72.7)	81 (61.4)
Body mass index (BMI), mean (SD), kg/m^2^	27.8 (5.4)	29.1 (5.3)^a^
Comorbid condition, *n* (%)		
Cardiac	40 (33.1)	96 (72.7)
Malignancy	3 (2.5)	2 (1.5)
Diabetes mellitus	22 (18.2)	132 (100)
Hepatic	4 (3.3)	4 (3.0)
Renal	3 (2.5)	5 (3.8)
Respiratory	10 (8.3)	117 (88.6)
Vascular	19 (15.7)	18 (13.6)
Lesion size, median (range), (cm^2^)	32 (2-638)	15 (1–300)^b^
Depth of lesion, median (range), (mm)	30 (4–150)	14 (0–50)^b^
Deepest tissue layer infected, *n* (%)		
Dermis	0 (0.0)	8 (6.1)^b^
Subcutaneous fat	57 (47.1)	10 (7.7)
Fascia, muscle, or deeper	64 (52.9)	112 (86.2)
Cellulitis		
No	43 (35.5)	45 (34.6)^c^
Yes	78 (64.5)	85 (65.4)
Smoking status		
Non/passive	64 (52.9)	92 (69.7)
Active	57 (47.1)	40 (30.3)
Alcohol consumption		
Abstinent	54 (44.6)	77 (58.3)
Any	67 (55.4)	55 (41.7)
PEDIS infection score		
2	N.A.	19 (15.6)^d^
3	N.A.	97 (79.5)
4	N.A.	6 (4.9)
University of Texas wound classification		
Grade 0, infected	N.A.	1 (0.8)^d^
Grade 0, ischemic	N.A.	0 (0.0)
Grade I, infected	N.A.	0 (0.0)
Grade I, ischemic	N.A.	18 (14.8)
Grade II, infected	N.A.	12 (9.8)
Grade II, ischemic	N.A.	52 (42.6)
Grade III, infected	N.A.	5 (4.1)
Grade III, ischemic	N.A.	34 (27.9)
Glycosylated hemoglobin (HbA1c,) mean (SD), %	N.A.	7.1 (2.5)^d^
Peripheral arterial diseasee		
No	N.A.	21 (16.4)^f^
Yes	N.A.	107 (83.6)
Peripheral neuropathy		
Vibration perception—negative	N.A.	67 (51.5)^c^
Vibration perception—positive	N.A.	63 (48.5)
Light pressure—negative	N.A.	70 (54.7)^e^
Light pressure—positive	N.A.	58 (45.3)

SD, standard deviation

^a^BMI was not available for 1 patient with DFI (*n* = 131)

^b^In 2 patients with DFI, lesion size/depth of lesion was not evaluated (*n* = 130)

^c^In 2 patients with DFI, cellulitis/vibration perception test was not available (*n* = 130)

^d^In 10 patients with DFI, PEDIS/University of Texas classification/HbA1c was not available (*n* = 122)

^e^Defined as ankle-brachial index (ABI) < 0.9 and/or foot pulses barely or not palpable; foot pulses as barely or not palpable were examined in the dorsalis pedis and posterior tibialis arteries

^f^In 4 patients with DFI, peripheral arterial disease/light pressure test was not available (*n* = 128)

### Patients with major abscesses

#### Analysis of severity of major abscesses based on lesion size as a continuous variable

In supplementary Table 4, the univariate analysis of association of polymorphisms and selected variables is presented, with surface lesion size as a continuous variable. In the dominant model, lesion size was significantly different comparing males and females (*p* = .021) and in patients with higher BMI (*p* = .009). Patients with any comorbidities significantly differed from those without comorbidities in terms of lesion size (*p* = .001). Lesion size as a continuous variable was not significantly associated (at .05 significance level) with any SNP, nor with lifestyle factors. These findings were confirmed in the recessive model analysis.

In Table 2, the linear regression analysis of association between polymorphisms and selected variables with lesion size as a continuous variable is presented. All variables were included in the initial model, after which backward selection was applied. A log transformation on lesion size was performed to better conform to normality. Age (*p* = .020), gender (*p* = .011), and BMI (*p* = .007) were associated with lesion size. More specifically, younger age, female gender, and higher BMI were associated with a larger lesion size. The presence of any comorbidities was also associated with larger lesion size (*p* = .001). Presence of the hetero- or homozygous allelic variant *TLR6* P249S decreased lesion size by 0.6 cm^2^ on average (*p* = .033). No other polymorphisms or lifestyle factors were significantly associated with lesion size (data not shown). In the recessive model analysis the association between lesion size and age (*p* = .011), gender, (*p* = .016), BMI (*p* = .006), comorbidities (*p* < .001), and TLR6 P249S (*p* = .025) was confirmed. In addition, in the recessive model, the presence of the WT and heterozygous allelic variant *TLR1* R80T decreased lesion size by 0.16 cm^2^ on average (*p* = .018).

**Table 2:** Linear regression after log transformation: factors influencing lesion size (continuous) (abscess) (*n* = 121)

		Dominant model		Recessive model	
Variable		Estimate (SE)	*p* value	Estimate (S.E.)	*p* value
Intercept		2.595 (0.706)		3.157 (1.218)	
Age		− 0.023 (0.010)	.020	− 0.024 (0.009)	.011
Gender – Female		0.683 (0.265)	.011	0.656 (0.269)	.016
BMI		0.060 (0.022)	.007	0.062 (0.022)	.006
Comorbidities	1 or 2	1.035 (0.308)	.001	1.134 (0.304)	< .001
	More than 2	1.871 (0.522)	.001	2.025 (0.514)	< .001
*TLR6* P249S—het/homo (D)—WT/het (R)		− 0.509 (0.236)	.033	0.997 (0.438)	.025
*TLR1* R80T—het/homo (D)—WT/het (R)		ns	ns	− 1.830 (0.764)	.018

SE, standard error; BMI, body mass index; het/homo, heterozygous and homozygous; WT/het, wild-type and heterozygous; (D), dominant model analysis; (R), recessive model analysis; ns, not significant

### Patients with diabetic foot infections

#### Analysis of severity of diabetic foot infections based on PEDIS infection score

In supplementary Table 5, the univariate analysis of association of polymorphisms and selected variables with PEDIS is presented. PEDIS was not significantly associated with any SNP, nor with any demographic or lifestyle factors. All variables were included in the initial model for multiple logistic regression analysis, after which backward selection was applied. No polymorphisms nor demographic or lifestyle factors were significantly associated with PEDIS (data not shown).

#### Analysis of severity of diabetic foot infections based on lesion size as a continuous variable

Supplementary Table 6 presents the univariate analysis of the association of polymorphisms and selected variables with lesion size as a continuous variable. Using the dominant model, DFI patients significantly differed in lesion size depending on the presence of comorbidities (*p* = .022), which was confirmed in the recessive model analysis. Lesion size as a continuous variable significantly differed for patients hetero- or homozygous for the allelic variant *TLR1* S248N and those with wild-type alleles (*p* = .047) in the dominant model. This difference in lesion size between patients WT- and heterozygous and patients homozygous for the allelic variant *TLR1* S248N was not found in the univariate recessive model analysis (*p* = .713).

In Table 3, the linear regression analysis of association between polymorphisms and selected variables with lesion size as a continuous variable is presented. All variables were included in the initial linear regression model, after which backward selection was applied. A log transformation on lesion size was performed to conform better to normality. No demographic or lifestyle factors were significantly associated with lesion size, in both the dominant and recessive model analysis. Only SNP *TLR1* S248N was associated with an increased lesion size. DFI patients hetero- or homozygous for SNP *TLR1* S248N had on average 2.4 cm^2^ larger lesions compared to patients with the wild type (*p* = .045) using the dominant model. No polymorphisms were significantly associated with lesion size using the recessive analysis.

**Table 3:** Linear regression after log transformation: factors influencing lesion size (continuous) (DFI) (*n* = 130)

	Dominant model		Recessive model	
Variable	Estimate (SE)	*p* value	Estimate (S.E.)	*p* value
Intercept	1.948 (0.417)		-	
*TLR1* S248N—het/homo(D)—WT/het (R)	0.884 (0.435)	.045	-	

SE, standard error; het/homo, heterozygous and homozygous; WT/het, wild-type and heterozygous; (D), dominant model analysis; (R), recessive model analysis

## Discussion

This study showed that polymorphisms in PRRs genes were associated with severity of infection in patients with major abscesses based on lesion size at clinical presentation. Patients hetero- or homozygous for the allelic variant *TLR6* P249S had smaller abscess lesions at clinical presentation and patients homozygous for the allelic variant *TLR1* R80T had larger abscess lesions. Additionally, the study showed the independent impact of several demographic factors on the severity of infection in patients with major abscesses. Patients who were younger, female, have higher BMI, or have any comorbidity had a higher risk of developing larger abscess lesions. Lifestyle factors (i.e., smoking and alcohol consumption) did not affect clinical severity as defined by lesion size in patients with major abscesses. For patients with DFI, the PEDIS infection score was chosen as a measure for infection severity but none of the tested variables was significantly associated with higher PEDIS, and thus a more severe infection. However, the study showed that PRRs genes and demographic factors, but not lifestyle factors, were associated with lesion size, as a surrogate measure for DFI severity. Patients with DFIs hetero- or homozygous for *TLR1* S248N wild-type alleles had a higher risk of developing larger lesions.

Our findings on the effect of polymorphisms and patient characteristics or lifestyle factors on the severity of major abscesses and DFIs are in accordance with previously found associations between polymorphisms in *TLR1* and *TLR6* genes, and susceptibility to cSSSIs in an analysis comparing patients with geographically matched healthy controls [8]. Indeed, presence of the polymorphisms *TLR1* S248N, *TLR6* P249S, and to some extent *TLR1* R80T was linked to higher susceptibility to cSSSIs, which may be attributed to a significantly lower IL-6 secretion [8]. Decreased IL-6 secretions by polymorphisms in TLR genes could result in a weakened immune response to infection, and thus in less abscess formation. On the other hand, elevated baseline values of IL-6 in DM patients could lead to tolerance after stimulation by pathogens [19]. Both this tolerance and the *TLR1* N248S polymorphism, resulting in lower IL-6 production, could contribute to larger DFI lesions [19]. Interestingly, *TLR1* N248S was previously shown to be associated with increased susceptibility to different infectious diseases including cSSSIs, tuberculosis, leprosy, aspergillosis, candidemia [20], Q fever [21], and malaria [22]. In addition, polymorphisms of *TLR1* N248S have been associated with differences in severity of malaria infections [23]. Similarly, *TLR 6* P249S was previously associated with susceptibility to tuberculosis, aspergillosis and malaria [20]. Altogether, these previous findings and ours suggest functional immunophenotypes for *TLR1* N248S and *TLR6* P249S for infectious diseases. Strikingly, polymorphisms in *TLR2* and *TLR10* genes previously found to be associated with increased susceptibility to cSSSIs [8, 9] did not affect clinical severity as defined by lesion size in patients with major abscesses and DFIs in this study.

In this study, we also observed that age has a protective role in containing the size of lesion in patients with major abscesses. Furthermore, females with major abscesses were more likely to have a larger lesion size, thus pointing to a more severe inflammation. BMI was a significant risk factor for larger lesions in all performed analyses for patients with abscesses. The presence of comorbidities was also associated with larger abscess lesions. These factors are known to be associated with immune activity (see “Methods“).

One of the strengths of this work is the quality of the clinical data. The RELIEF trial was a prospective study, where participants were immediately asked to participate in this genetic substudy. In addition, the lesion size measurements by clinician investigators were validated by a DRC using photographs [10]. The direct combination of a registration study with large cohorts, such as the RELIEF trial, with studies evaluating immunological and genetic factors associated with the disease was very beneficial as it allowed for reduced research efforts and costs while still yielding adequate numbers of participants. Genetic studies do require a large number of patients, as the prevalence of alleles in the population cannot be predicted and can be rare in the study population. Associations between SNPs and disease are highly influenced by, e.g., disease prevalence, disease allele frequency, and effect size of the genetic variants.

Limitations of this study include the selection of only 13 SNPs in PRRs genes and the signaling adaptor TIRAP. SNPs in genes coding for TLR1, TLR2, TLR6, and TLR10 were selected based on previously described associations with human disease or known functional effects on gene expression or protein function. [8]. A genome-wide association study could be considered, to screen for more polymorphisms and correlate those to disease severity, but the number of patients available would limit the strength of this approach [24]. A second limitation is the assessment of infection severity. Size of the primary lesion at clinical presentation was chosen as the only indicator as pain intensity [13] was not measured in the original trial. This is in accordance with the most recent FDA guidance defining ABSSSI by lesion size [4]. However, the size of lesions may also illustrate patients’ immune response to the infection. In addition to lesion size, in our study, severity of infection in the DFI population was also assessed using the PEDIS infection classification. No associations between variables and PEDIS were found, even though PEDIS is the most used indicator of severity of DFI lesions. However, this could be due to the low number of patients with a PEDIS score of 2 in this study.

In conclusion, this study showed that polymorphism *TLR6* P249S plays a protective role, in contrast to polymorphism *TLR1* R80T, in the severity of major abscesses, defined as lesion size at clinical presentation. Polymorphism *TLR1* S248N was associated with severity of DFIs, defined as lesion size at clinical presentation. Furthermore, age, gender, BMI, and comorbidities were associated with abscess severity. Future studies should focus on validating our results in larger cohorts. Furthermore, the predictive value of these SNPs and demographic factors in the prognosis of patients should be evaluated.

## Electronic supplementary material

ESM 1 (DOCX 50.4 kb)
