# Supplementary material for: Lesion size is associated with genetic polymorphisms in TLR1, TLR6, and TIRAP genes in patients with major abscesses and diabetic foot infections
Source: Eur J Clin Microbiol Infect Dis. 2019 Nov 30;39(2):353–60. doi: 10.1007/s10096-019-03732-7 (PMC7010613; doi:10.1007/s10096-019-03732-7)
Supplement: Supplementary file 1 — (DOCX 50.4 kb) [file 10096_2019_3732_MOESM1_ESM.docx]

**Supplementary Material**

**Supplementary Table 1**: Genotyped nonsynonymous missense single-nucleotide polymorphisms (SNPs) in genes coding for pattern-recognition receptors and the signaling adaptor TIRAP.

| **Gene** | **SNP ID** | **Mutation*** | **Nucleotide Change** | **Amino Acid Change** |
| --- | --- | --- | --- | --- |
| *TLR1* | rs4833095 | Missense | C>T | S248N˭ |
| *TLR1* | rs5743611 | Missense | G>C | R80T |
| *TLR2* | rs5743704 | Missense | C>A | P631H˭ |
| *TLR2* | rs5743708 | Missense | G>A | R753Q |
| *TLR 4* | rs4986790 | Missense | A>G | D299G |
| *TLR 4* | rs4986791 | Missense | C>T | T399I |
| *TLR6* | rs5743810 | Missense | C>T | P249S˭ |
| *TLR10* | rs11096955 | Missense | A>C | I369L^#^ |
| *TLR10* | rs4129009 | Missense | A>C | I775L^#^ |
| *TLR10* | rs11466653 | Missense | T>C | M326T |
| *TLR10* | rs11096957 | Missense | A>C | N241H^#^ |
| *NOD2* | rs2066842 | Missense | C>T | P268S |
| *TIRAP* | rs8177374 | Missense | C>T | S180L |

TIRAP: Toll-interleukin 1 receptor domain-containing adaptor protein.

* The first nucleotide (and corresponding amino acid) is ancestral and is therefore considered the wild-type allele.

˭ Associated with an increased susceptibility to complicated skin and skin structure infections (cSSSIs). # Associated with decreased susceptibility to cSSSIs.

**Supplementary Table 2:** Microbiological characteristics of patients with major abscesses (n=121) or Diabetic Foot Infection (DFI) (n=130).

|  | Abscesses | | DFI | |
| --- | --- | --- | --- | --- |
|  | **Small Area (≤32cm^2^)**  **(n=64)** | **Large Area (>32cm^2^) (n=57)** | **Mild PEDIS infection grade 2 (n=19)** | **Moderate/severe PEDIS infection grade 3+4 (n=103)** |
| **Cultures obtained** |  |  |  |  |
| Abscess | 60 (93.7) | 50 (87.7) | 0 (0.0) | 0 (0.0) |
| Wound | 0 (0.0) | 0 (0.0) | 19 (100) | 98 (95.1) |
| None | 4 (6.3) | 7 (12.3) | 0 (0.0) | 5 (4.9) |
|  |  |  |  |  |
| **Growth** |  |  |  |  |
| Yes | 48 (80.0) | 40 (80.0) | 15 (78.9) | 91 (92.9) |
| No | 12 (20.0) | 10 (20.0) | 4 (21.1) | 7 (7.1) |
|  |  |  |  |  |
|  |  |  |  |  |
| **Microorganisms identified** | 75 | 60 | 27 | 179 |
| Polymicrobial | 20 (41.7) | 14 (35.0) | 10 (66.7) | 55 (60.4) |
| *S. aureus* | 32 (42.7) | 17 (28.3) | 10 (37.1) | 58 (32.4) |
| MSSA | 30 (93.8) | 16 (94.1) | 9 (90.0) | 46 (79.3) |
| MRSA | 2 (6.2) | 1 (5.9) | 1 (10.0) | 12 (20.7) |
| Streptococci | 10 (13.3) | 7 (11.7) | 5 (18.5) | 34 (19.0) |
| Enterococci | 8 (10.7) | 5 (8.4) | 5 (18.5) | 36 (20.1) |
| Enterobacteriaceae | 14 (18.7) | 17 (28.3) | 2 (7.4) | 29 (16.2) |
| Bacteroides spp | 5 (6.7) | 8 (13.3) | 1 (3.7) | 3 (1.7) |
| Anaerobes | 5 (6.7) | 4 (6.7) | 2 (7.4) | 10 (5.6) |
| Other | 1 (1.2) | 2 (3.3) | 2 (7.4) | 9 (5.0) |
| MSSA: Methicillin-sensitive *Staphylococcus aureus*; MRSA: Methicillin-resistant *Staphylococcus aureus*; Spp: species | | | | |

**Supplementary Table 3:** Distribution of pattern-recognition receptor (PRR) and signaling adaptor TIRAP genotypes in 121 patients with major abscesses and 132 patients with Diabetic Foot Infection (DFI).

| **Polymorphism** | **Wild-type, N(%)** | **Heterozygous, N(%)** | **Homozygous N(%)** |
| --- | --- | --- | --- |
| ***TLR1* R80T** | GG | GC | TT |
| Patients with major abscesses | 90 (74.4) | 28 (23.1) | 3 (2.5) |
| Patients with DFI | 93 (70.5) | 37 (28.0) | 2 (1.5) |
| ***TLR1* S248N** | CC | CT | TT |
| Patients with major abscesses | 10 (8.3) | 43 (35.5) | 68 (56.2) |
| Patients with DFI | 11 (8.3) | 56 (42.4) | 65 (49.3) |
| ***TLR6* P249S** | CC | CT | TT |
| Patients with major abscesses | 50 (41.3) | 62 (51.2) | 9 (7.5) |
| Patients with DFI | 61 (46.2) | 49 (37.1) | 22 (16.7) |
| ***TLR 4* D299G** | AA | AG | GG |
| Patients with major abscesses | 108 (89.3) | 13 (10.7) | 0 |
| Patients with DFI | 116 (87.9) | 15 (11.4) | 1 (0.7) |
| ***TLR 4* T399I** | CC | CT | TT |
| Patients with major abscesses | 108 (89.3) | 13 (10.7) | 0 |
| Patients with DFI | 116 (87.9) | 15 (11.4) | 1 (0.7) |
| ***TLR2* R753Q** | GG | GA | AA |
| Patients with major abscesses | 114 (94.2) | 7 (5.8) | 0 |
| Patients with DFI | 126 (95.5) | 6 (4.5) | 0 |
| ***TLR2* P631H** | CC | CA | AA |
| Patients with major abscesses | 110 (90.9) | 11 (9.1) | 0 |
| Patients with DFI | 117 (88.6) | 15 (11.4) | 0 |
| ***TLR10* M326T** | TT | TC | CC |
| Patients with major abscesses | 115 (95.0) | 6 (5.0) | 0 |
| Patients with DFI | 123 (93.2) | 9 (6.8) | 0 |
| ***TLR10* N241H** | AA | AC | CC |
| Patients with major abscesses | 49 (40.5) | 57 (47.1) | 15 (12.4) |
| Patients with DFI | 47 (35.6) | 58 (43.9) | 27 (20.5) |
| ***TLR10* I775L** | AA | AC | CC |
| Patients with major abscesses | 83 (68.6) | 34 (28.1) | 4 (3.3) |
| Patients with DFI | 83 (62.9) | 42 (31.8) | 7 (5.3) |
| ***TLR10* I369L** | AA | AC | CC |
| Patients with major abscesses | 49 (40.5) | 57 (47.1) | 15 (12.4) |
| Patients with DFI | 47 (35.6) | 58 (43.9) | 27 (20.5) |
| ***NOD2* P268S** | CC | CT | TT |
| Patients with major abscesses | 63 (52.1) | 44 (36.3) | 14 (11.6) |
| Patients with DFI | 79 (59.8) | 45 (34.1) | 8 (6.1) |
| ***TIRAP* S180L** | CC | CT | TT |
| Patients with major abscesses | 87 (71.9) | 29 (24.0) | 5 (4.1) |
| Patients with DFI | 101 (76.5) | 27 (20.5) | 4 (3.0) |

**Supplementary Table 4.** Univariate analysis: association of selected variables with lesion size of abscesses (continuous) (n=121).

|  |  | **Dominant model** | | **Recessive model** | |
| --- | --- | --- | --- | --- | --- |
| **Variable** |  | **Mean area (±SD)** | ***p*-value** | **Mean area (±SD)** | ***p*-value** |
| Age |  |  | .258 |  | .258 |
| Gender |  |  | .021 |  | .021 |
|  | Male | 82.4 (±134.4) |  | 82.4 (±134.4) |  |
|  | Female | 111.6 (±126.3) |  | 111.6 (±126.3) |  |
| BMI |  |  | .009 |  | .009 |
| Comorbidity | None  1-2  >2 | 74.0 (±136.6)  103.3 (±134.0)  137.1 (±78.2) | .001 | 74.0 (±136.6)  103.3 (±134.0)  137.1 (±78.2) | .001 |
| Smoker |  |  | .168 |  | .168 |
|  | Non/Passive | 103.2 (±140.0) |  | 103.2 (±140.0) |  |
|  | Active | 75.9 (±123.0) |  | 75.9 (±123.0) |  |
| Alcohol consumption |  |  | .878 |  | .878 |
|  | Abstinent | 103.0 (±154.0) |  | 103.0 (±154.0) |  |
|  | Any | 80.2 (±112.2) |  | 80.2 (±112.2) |  |

SD: standard deviation; BMI: body mass index

**Supplementary Table 5**. Univariate analysis: association of selected variables with severity of cSSSI (PEDIS infection score), DFI population (N=122).

| **Variable** |  | **Mild PEDIS infection grade 2**  **(n=19)** | **Moderate/ Severe PEDIS infection grade 3+4**  **(n=103)** | **OR (95% CI)** | **p-value** |
| --- | --- | --- | --- | --- | --- |
| Age |  | 57 (±9.3) | 61 (±10.2) |  | .150 |
| Sex |  |  |  | 0.505 (0.188-1.353) | .169 |
|  | Male | 9 (7.4%) | 66 (54.1%) |  |  |
|  | Female | 10 (8.2%) | 37 (30.3%) |  |  |
| BMI |  | 29.6 (±5.2) | 28.9 (±5.3) |  | .628 |
| Comorbidity | Only DM  DM+1  DM+2 or more | 1 (0.9%)  7 (5.7%)  11 (9.0%) | 7 (5.7%)  24 (19.7%)  72 (59.0%) |  | .446 |
| PAD |  |  |  | 0.625 (0.131 – 2.973) | .735 |
|  | Normal | 2 (1.7%) | 16 (13.3%) |  |  |
|  | Abnormal | 17 (14.2%) | 85 (70.8%) |  |  |
| HbA1c |  |  |  | 1.709 (0.460 – 6.353) | .559 |
|  | ≤ 7% | 16 (13.1%) | 78 (63.9%) |  |  |
|  | > 7% | 3 (2.5%) | 25 (20.5%) |  |  |
| Smoker |  |  |  | 4.188 (0.915-19.182) | .057 |
|  | Non/Passive | 17 (13.9%) | 69 (56.6%) |  |  |
|  | Active | 2 (1.6%) | 34 (27.9%) |  |  |
| Alcohol consumption |  |  |  | 1.928 (0.646-5.758) | .234 |
|  | Abstinent | 14 (11.5%) | 61 (50.0%) |  |  |
|  | Any | 5 (4.1%) | 42 (34.4%) |  |  |

BMI: body mass index; PAD: peripheral artery disease; HbA1c: Glycated haemoglobin; PEDIS: perfusion extent/size, depth/tissue loss. infection and sensation.

**Supplementary Table 6:** Univariate analysis: association of selected variables with lesion area of DFI (continuous) (n=130).

|  |  | **Dominant model** | |  | **Recessive model** | |
| --- | --- | --- | --- | --- | --- | --- |
| **Variable** |  | Mean area (±SD) | | p-value | Mean area (±SD) | p-value |
| Age |  |  | .904 | |  | .904 |
| Sex |  |  | .077 | |  | .077 |
|  | Male | 39.4 (±49.9) |  | | 39.4 (±49.9) |  |
|  | Female | 38.7 (±66.5) |  | | 38.7 (±66.5) |  |
| BMI |  |  | .738 | |  | .738 |
| Comorbidity | Only DM  DM+1  DM+2 or more | 29.9 (±41.6)  64.7 (±70.0)  30.8 (±49.6) | .022 | | 29.9 (±41.6)  64.7 (±70.0)  30.8 (±49.6) | .022 |
| PAD |  |  | .636 | |  | .636 |
|  | Normal | 37.9 (±62.0) |  | | 37.9 (±62.0) |  |
|  | Abnormal | 38.4 (±54.6) |  | | 38.4 (±54.6) |  |
| HbA1c |  |  | .724 | |  | .724 |
|  | ≤ 7% | 39.2 (±54.0) |  | | 39.2 (±54.0) |  |
|  | > 7% | 38.9 (±65.2) |  | | 38.9 (±65.2) |  |
| Smoker |  |  | .675 | |  | .675 |
|  | Non/Passive | 41.9 (±60.5) |  | | 41.9 (±60.5) |  |
|  | Active | 32.6 (±45.8) |  | | 32.6 (±45.8) |  |
| Alcohol consumption |  |  | .701 | |  | .701 |
|  | Abstinent | 42.1 (±62.2) |  | | 42.1 (±62.2) |  |
|  | Any | 34.8 (±47.1) |  | | 34.8 (±47.1) |  |
| *TLR1* S248N |  |  | .047 | |  | .713 |
|  | WT | 18.5 (±26.7) |  | | 33.7 (±52.3) |  |
|  | Het/Homo | 41.0 (±58.2) |  | | 44.2 (±60.1) |  |

SD: standard deviation; BMI: body mass index; PAD: peripheral artery disease; HbA1c: Glycated haemoglobin; WT: wild type; Het/Homo: heterozygous and homozygous

Excluded:

infected wound infection (n=45)

infected ischemic ulcers (n=20)

Excluded :

not European ancestry (n=2)

not of Eastern European ethnicity (n=69)

813 patients with cSSSI (Gyssens et al. 2011)

424 patients excluded due to absence of invitation or refusal

389 patients eligible for genetic substudy (Stappers et al. 2014)

318 patients included for genetic substudy

121 patients with abscess

132 patients with DFI

**Supplementary Figure 1.** Flowchart of patients enrolled in this study from the RELIEF cohort (n=813).

cSSSI: complicated skin and skin structure infections; DFI: Diabetic Foot Infection.

References:

Gyssens, I. C., Dryden, M., Kujath, P., Nathwani, D., Schaper, N., Hampel, B., ... & Arvis, P. (2011). A randomized trial of the efficacy and safety of sequential intravenous/oral moxifloxacin monotherapy versus intravenous piperacillin/tazobactam followed by oral amoxicillin/clavulanate for complicated skin and skin structure infections. Journal of antimicrobial chemotherapy, 66(11), 2632-2642.

Stappers, M. H., Thys, Y., Oosting, M., Plantinga, T. S., Ioana, M., Reimnitz, P., ... & Gyssens, I. C. (2014). TLR1, TLR2, and TLR6 gene polymorphisms are associated with increased susceptibility to complicated skin and skin structure infections. The Journal of infectious diseases, 210(2), 311-318.
